# Access to medicines through global health diplomacy

**DOI:** 10.34172/hpp.2023.05

**Published:** 2023-04-30

**Authors:** Vijay Kumar Chattu, Bawa Singh, Sanjay Pattanshetty, Srikanth Reddy

**Affiliations:** ^1^Department of Occupational Science & Occupational Therapy, Temerty Faculty of Medicine, University of Toronto, Toronto, ON M5G 1V7, Canada; ^2^United Nations University- Institute on Comparative Regional Integration Studies (UNU-CRIS), 8000 Brugge, Belgium; ^3^Center for Evidence-Based Diplomacy, Global Health Research and Innovations Canada (GHRIC), Toronto, ON, Canada; ^4^Department of South and Central Asian Studies, School of International Studies, Central University of Punjab, Bathinda-India; ^5^Department of Global Health Governance, Prasanna School of Public Health, Manipal Academy of Higher Education, Manipal, India; ^6^School of Epidemiology and Public Health, University of Ottawa, Ottawa, Canada; ^7^Bruyere Research Institute, Ottawa, Canada

**Keywords:** Health equity, Access to medicines, Sustainable development goals, Global health, Diplomacy, Security measures, COVID-19, Vaccines, Pharmaceuticals

## Abstract

The World Health Organisation (WHO) emphasizes that equitable access to safe and affordable medicines is vital to attaining the highest possible standard of health by all. Ensuring equitable access to medicines (ATM) is also a key narrative of the Sustainable Development Goals (SDGs), as SDG 3.8 specifies "access to safe, effective, quality and affordable essential medicines and vaccines for all" as a central component of universal health coverage (UHC). The SDG 3.b emphasizes the need to develop medicines to address persistent treatment gaps. However, around 2 billion people globally have no access to essential medicines, particularly in lower- and middle-income countries. The states’ recognition of health as a human right obligates them to ensure access to timely, acceptable, affordable health care. While ATM is inherent in minimizing the treatment gaps, global health diplomacy (GHD) contributes to addressing these gaps and fulfilling the state’s embracement of health as a human right.

## Introduction

 The burning issue of access to medicines (ATM) is a fundamental component to be resolved in order for the right to health to be fully realized.^1^ Equitable access to medical and health products is a priority worldwide. Therefore, the availability, accessibility, acceptability, and affordability of these products of assured quality must be addressed to achieve the United Nations’ Sustainable Development Goals (SDGs), especially target 3.8.^2^ Despite the progress made globally, millions of people lack ATM in the developing countries due to the hindrances that block access to quality medicines. From a human rights perspective, ATM is intrinsically linked with the principles of “equality and non-discrimination”, “transparency”, “participation”, and “accountability”. Universal health coverage (UHC) ensures that globally, people have the continuous access to high-quality essential medical and health services, secure, reliable, affordable essential medicines and vaccines, and also financial security.^3^ Because developing countries have the greatest demand and the least ATM, there is still an inherent connection between poverty and the achievement of the right to health. This undermines human dignity and the foundation of all human rights, particularly the rights of all people to life, health, and development. ATM is an inter-sectoral, multi-stakeholder and inter-disciplinary issue.

 According to Health Diplomats, “*Health Diplomacy is the chosen method for interaction between stakeholders engaged in public health and politics for the purpose of representation, cooperation, resolving disputes, improving health systems, and securing the right to health for vulnerable populations.*”^4^ Given this context, health diplomacy has a potential role in addressing the challenges and filling the gaps created by the inaccessibility to essential medicines and drugs. In this challenging period of the COVID-19 pandemic, there have been reports everywhere that there was a disproportionate impact on vulnerable groups. People living in poverty, children, and others in vulnerable situations such as the elderly, people with disabilities, women, girls, other groups such as migrants, minorities and LGBTIQ (people who have identified themselves as lesbian, gay, bisexual, transgender, intersex, or questioning)were deprived of their liberties and access to healthcare services. To address these challenges, there is a great need for a global governance mechanism that could bring policies and hard/soft law instruments with high-level political commitments. As highlighted by various researchers, global health diplomacy (GHD) is a soft power that has the potential to negotiate various global policies for promoting health as a public good. According to Kickbusch, GHD is a “*multi-level, multi-actor negotiation process that shapes and manages the global policy environment for health*.”^5^ Through GHD, global collaboration and actions can be adopted to increase universal access to health as a basic human right, including ATM, medical products and therapies, assistive and advanced technologies and palliative care. Some recent examples of ongoing and successful GHD are shown in Table 1.

**Table 1:** Examples of some ongoing and successful outcomes of global health diplomacy

**Item No.**	**Example **
1	Global Pandemic Treaty/ International Agreement (in the negotiation phase)
2	Development of COVAX Facility (established and delivering results)
3	Health Diplomacy by India and South Africa at WTO for a TRIPS-waiver for COVID-19 medicines, therapeutics, and diagnostics (awaiting final decision)
4	Development and ratification of the African Medicines Agency
5	Framework Convention on Tobacco Control
6	Port of Spain Summit Declaration for the prevention of non-communicable diseases

Abbreviations: WTO, World Trade Organization; TRIPS, Agreement on Trade-Related Aspects of Intellectual Property Rights.

## Health equity and access to medicines

 The World Health Organization (WHO) declared COVID-19 as a pandemic on March 11, 2020, after the detection of first cases in Wuhan, China in December 2019. In the wake of its declaration as a global pandemic, COVID-19 has devastated numerous nations and overwhelmed their healthcare systems. Globally, as of December 19, 2022, around 649 038 437 confirmed cases of COVID-19, including 6 649 812 cumulative deaths and 64 631 new cases were identified in the last 24 hours.^6^ The situation becomes more critical when the ATM is limited despite several measures were put in place through global governance mechanisms. This has made COVID-19 the most significant global health crisis since the influenza pandemic of 1918.^7^ The International Labour Organization (ILO) says that the number of lost working hours in 2020 was equal to 255 million full-time jobs, which cost $3.7 trillion in lost labor income. It has sent shockwaves through the global economy.^8^ Many countries have experienced a second, third or fourth wave of outbreaks of this viral illness. As the COVID-19 cases worldwide has grown exponentially, vaccine development had to be completed by the end of 2020.

 Out of the 7.8 billion people in the world, 2.3 to 2.6 billion live in low- and middle-income countries. About 333 million people in the upper middle lived in countries with an upper middle income. On the other hand, about 972 million people live in countries with high incomes. But the irony is that the poorest 80% of people only have access to less than 20% of the total wealth, productive capacity, etc. Tedros Adhanom Ghebreyesus (Director General of the WHO) said in his opening remarks, “Vaccine equity is the problem of our time.” “We are also not doing well”.^9^

 According to the research findings by various scholars, there were huge differences and inequalities that happened during the COVID-19 pandemic, for example, in testing for infections, hospitalizations, and deaths.^10,11^ A recent study that looked at data up to end of March, 2021 (about 4 months after the first public COVID-19 vaccination, which had happened on December 6, 2020), found that the global Gini coefficient was 0.88 for COVID-19 vaccinations.^12^ A study by Tatar et al,^12^ showed that the distribution of COVID-19 vaccinations did not get any better, and also differences between vaccinations for COVID-19 have widened by December 7, 2021.

 The inequality between continents was slightly less severe (0.61), but the inequalities within continents were very severe. Europe, on the other hand, has a bit less inequality (0.73). Also, 59.3% of the world’s people live in Asia, and even though 67.8% of the world’s vaccines were given to Asian countries, they were not all given the same amount (the Gini coefficient was 0.86 on December 7, 2021). Oceania had a lot of the same problems with vaccines (0.88). On the other hand, even though Africa had 17.5% of the world’s population, it only used 3.1% of the world’s vaccines. This made the distribution of vaccines in African continent less unfair than in Asia (the Gini coefficient as of 7^th^ December 2021, was 0.73). However, Europe and South America got about the same number of COVID-19 vaccines as the populations and the distributions in these regions were not as uneven as in Asia, Oceania, or North America.

## Barriers to access to medicines

 Given COVID-19’s multilateral impacts, health equity has become one of the major challenges for global health governance. Against this background, accessibility of medicine has remained limited, given several barriers for the developed world in general and developing countries in particular. The patents grant one of the critical barriers to the same. It is exclusively given for 20-year territorial rights to a product or process. The patent owner can prevent the product’s manufacture, use, sale, import, or distribution.^13^ Patent protection gives pharmaceutical companies monopolies on drugs and processes.^14^

 Data extending exclusivity periods can also affect drug delivery to developing countries, especially generics. Some say data exclusivity reduces generic drug availability. Many contend that pharmaceutical firms work to secure data exclusivity in order to safeguard innovative medications and maintain their monopolies.^14^ Cost is another barrier to medicine equity. Pharmaceutical companies in some countries have complete control over the pricing of their patented product. As a result, the company has entire control over the medication’s cost, which is determined by the price level the company believes best reflects their capacity to produce and the intended level of profit. Purchasers have little influence over the price that is set.^15^ In addition to leaving the buyer impacted, this drastic price hike also creates inequalities in the ability of different socioeconomic groups to obtain basic necessities.^16^

 The developing countries have remained the most affected ones. Many argue that the production of generic brands in developing nations increases competition and is necessary to close the global drug gap.^17^ According to multiple sources, the demand for extra safeguards such as market and data exclusivity impedes low-income countries’ ability to manufacture and produce generic pharmaceuticals. However, low-income nations frequently lack the necessary infrastructure to produce generic brands. The Agreement on Trade-Related Aspects of Intellectual Property Rights (TRIPS) is one of the most critical barriers for ATM. On the part of some scholars, it is argued that the TRIPS Agreement used to have the most significant impact on the pharmaceutical industry and ATM.^18^ It is also argued that the TRIPS agreement had a negative effect on the generic drug industries in several countries. On the contrary, it is argued that the agreement is interpretable. In the event of a national emergency, a TRIPS clause allows for compulsory licensing, which allows for the creation of generic versions of patented medications at market-determined pricing.^15^

## Access to medicines through global health diplomacy

 The GHD has played a very constructive role in the ATM. The WHO has initiated several efforts to combat the COVID-19 pandemic. It started the Solidarity Trial to investigate possible treatments, the United Nations COVID-19 Supply Chain Task Force, and the “COVID-19 Solidarity Response Fund” to raise money for the response. As highlighted by Chattu et al, international organizations have taken the lead role in forming global alliances to accelerate vaccine research and development (R&D).^19^ The multilaterals such as World Bank and WHO, Bill and Melinda Gates Foundation (BMGF), and other international non-governmental organizations (INGOs) have raised $8.1 billion and introduced the WHO COVID-19 Vaccines Global Access (COVAX) plan for a fair and equitable distribution of licensed vaccines. The Coalition for Epidemic Preparedness Innovations (CEPI) has also established a second fund of $US 2 billion from a global partner for expedited research and clinical testing. Other nations such as Belgium, Canada, Germany, Norway, the Netherlands, Switzerland, and the UK, as well as charitable organizations such as BMGF, have contributed approximately $915 million and $250 million, respectively, to CEPI research and public education support for promoting COVID-19 vaccine acceptance. Concurrently, the Global Research Collaboration for Infectious Disease Preparedness (GLoPID-R) and the International Severe Acute Respiratory and Emerging Infection Consortium also collaborate on research related to COVID-19 and for vaccine distribution. Representatives from 52 nations, including 35 heads of state from the G7 and G20, participated in a virtual summit to support the Global Alliance for Vaccines and Immunization (GAVI). For instance, the European Commission had contributed almost €80 million to CureVac.^19^

 The COVAX program is jointly led by the WHO, the GAVI, and the CEPI to speed up the production of COVID-19 vaccines and ensure that all countries have equal and fair access to them.^20^ Berkley argued that in the very beginning of the pandemics, it became clear that in addition to COVID-19 vaccines, we also needed to make sure that everyone in the world had access to them to put an end to this global crisis.^21^ This stimulated world leaders to demand a solution that would hasten the development and production of COVID-19 vaccines, as well as diagnostics and treatments, and ensure people in all countries have quick, fair, and equitable access to them. Today, COVAX is the remedy of choice. COVAX is the product of an extraordinary and singular global collaboration in which more than two-thirds of the world participated. As such, COVAX represents the best chance to end this pandemic’s acute phase as soon as possible.

 COVAX, coordinated by the GAVI, CEPI, and the WHO, has become an important instrument of global health diplomacy. The same has been a platform to support the development, manufacturing, and pricing of various COVID-19 vaccines. Once created, all participating countries, regardless of income level, will have equal access to these vaccines. The initial target is to have around 2 billion doses made available by December 2021, which should be sufficient to protect frontline healthcare workers, vulnerable patients, and high-risk individuals.^22^

 COVAX has demonstrated its efficacy as the global mechanism for equitable access to COVID-19 vaccines. Since February, it has distributed over 70 million vaccine doses to 126 countries and economies worldwide, from remote islands to conflict zones, managing the largest and most challenging vaccine rollout in history. The first doses of the COVID-19 vaccine were distributed to over 35 countries by the same. So far, COVAX has distributed over 1 billion doses of COVID-19 vaccines to around 144 participants.^23^

 There is a great scope for GHD to move things forward through global partnerships, negotiations, and emphasizing health security, which can only be achieved through equity. For example, African Medical Agency has recently evolved from health diplomacy and continues to encourage governments’ participation to contribute to the ATM.^24^Table 2 summarises the various high-level political declarations and roadmap for ATM.

**Table 2:** Summary of the political declarations and roadmap for access to medicines from 2000-till date

**Global organization/ Authority**	**Resolution/year**	**Topic/Area**
OHCHR	High Commissioner Report on COVID-19 vaccines (2022). Ref: A/HRC/49/35	“The human rights implications of the lack of affordable, timely, equitable, and universal access and distribution of COVID-19 vaccines.” The growing disparities across states underline the fact that vaccine delays have serious health impacts as well as fundamental human rights implications.
WHO	The 71^st^ WHA’s Director General’s Report Ref: (document A71/12). May 2018,	“Addressing global medicine and vaccine shortages and improving access”- “Road map for access to medicines, vaccines, and other related health products 2019–2023”
OHCHR report	A/HRC/47/23, May 2021	*“The State's crucial role in addressing pandemics and other health emergencies, as well as the socioeconomic fallout from doing so, in promoting sustainable development and the realization of all human rights”*
OHCHR report	Ref: A/HRC/46/19, March 2021	*“COVID-19 pandemic’s impact on the enjoyment of human rights worldwide, good practices and the areas of concern”*
HRC	HRC resolution Ref: 46/14 of 29 dated March 2021	“To ensure equitable, affordable, timely, and universal access vaccines for all countries in response to COVID-19”
HRC	HRC resolution Ref: 41/10 of 19 dated July 2019	“Access to medicines and vaccines in the context of the right of everyone to the enjoyment of the highest attainable standard of physical and mental health.”
UNGA	General Assembly’s resolution Ref: 73/3 dated 10 October 2018	“Political declaration of the high-level meeting of the General Assembly on the fight against tuberculosis.”
UNGA	General Assembly’s resolution Ref: 73/2 dated 10 October 2018	“Political declaration of the third high-level meeting of the General Assembly on the prevention and control of non-communicable diseases”
UNGA	General Assembly’s resolution Ref: 71/3 of 5 dated October 2016	“Political declaration of the high-level meeting of the General Assembly on antimicrobial resistance.”
UN Secretary-General	High-level Panel of United Nations Secretary-General dated September 2016	A web gage on access to medicines and the Panel’s report on “promoting innovation and access to health technologies”
HRC	HRC’s resolution Ref: 32/15 dated 1 July 2016	“Access to medicines in the context of the right of everyone to the enjoyment of the highest attainable standard of physical and mental health”
UNGA	General Assembly’s resolution Ref: 70/1 dated 25 September 2015	“Transforming our world: the 2030 Agenda for Sustainable Development.”
HRC Social Forum	A/HRC/29/44 of 2015	“Report of the 2015 Social Forum on Access to Medicines”
HRC Special Rapporteur	A/HRC/23/42), 1 May 2013	“Right of everyone to the enjoyment of the highest attainable standard of physical and mental health.”
HRC Special Rapporteur	A/63/263), 11 August 2008	“Human Rights Guidelines for Pharmaceutical Companies in relation to Access to Medicines”
Committee on Economic, Social and Cultural Rights	CESCR, General comments Ref: No. 17 (2006)	“The responsibilities of States parties to prevent high costs of essential medicines, plant seeds and food production from undermining the rights to health, food, and education.”
Committee on Economic, Social and Cultural Rights	CESCR, General comments Ref: No. 14 (2000)	Within the control of the State party, health facilities, goods, and services must be accessible to all without discrimination. The four overlapping dimensions of accessibility include Non-discrimination, accessibility in physical, Economic and information domains

Abbreviations: OHCHR, Commissioner of Office of the High Commissioner for Human Rights; WHO, World Health Organization; UNGA, United Nations General Assembly; HRC, Human Rights Council.

 Notwithstanding several efforts and initiatives, the issue of ATM has remained unsatisfactory. Rather, the gravity of the situation is becoming more and more critical, given intellectual property rights. In light of this, India and South Africa have emerged as the leading developing nations. As part of their global health diplomacy, India and South Africa jointly proposed (Ref: IP/C/W/669) before the WTO-TRIPS Council that the IPRs provisions be temporarily waived to hasten the development of drugs, vaccines, and diagnostics for the prevention, containment, and treatment of COVID19.^25^ Additionally, the proposal has a very wide scope, making almost every medical device used to diagnose, treat, or prevent COVID-19 are eligible for such a waiver.^26^ The same was further sparked when more than 350 organizations and activists from the global civil society urged WTO members to support the joint proposal from India and South Africa. According to the proposal’s provisions, nations must “waive off” patents, copyrights, and other intellectual property (IP) rights for the final products and the technologies that underlie them without fear of WTO charges or penalties for breaking trade laws.

 Additionally, many civil society organizations, the Joint United Nations Programme on HIV/AIDS (UNAIDS), Unitaid, MSF, academia, and researchers, supported the proposal.^27^ The South African and Indian proposal received the WHO’s full support along with other 14 nations. The Indian leadership/health authorities have realized that IP rights are becoming barriers for “scaling up production of test kit reagents, ventilator valves, N95 respirators, therapeutics, fluorescent proteins, other technologies used in the development of vaccines, etc.”^25^ However, due to the developed countries’ rejection and lack of agreement, the proposal was not accepted. Instead, three groups of WTO members have been created. On behalf of the African Group and least developed countries, Chad and Tanzania and a few other nations from South, Southeast, and South America supported the proposal. China, Costa Rica, Chile, Columbia, Jamaica, El Salvador, Senegal, and other nations in the second group welcomed the proposal but asked for more details. The developed nations that had flatly rejected the proposal included Brazil, Canada, Norway, the United Kingdom, the United States, Switzerland, and the European Union made up the third group.

 During the 12th WTO Ministerial Conference (12-17 June 2022), WTO members agreed to temporarily waive intellectual property patents on Covid-19 vaccines without obtaining the patent holder’s consent for five years.^28^ This will allow WTO members to manufacture COVID-19 vaccines more easily within their own countries. The original proposition that India and South Africa made in 2020 has been significantly watered down in the current agreement that they have reached. However, the rich pharmaceutical companies vigorously opposed this idea, arguing that intellectual property rights do not restrict access to COVID-19 vaccines and that removing patent protections send a negative message to researchers who quickly produced life-saving vaccines.

## Global health diplomacy, access to medicines and Sustainable Development Goals

 Even though the world was only a third of the way through the sustainable development agenda in December 2019, the COVID-19 crisis began in earnest, shattering many achievements and posing challenges to global health, social, economic, and developmental aspects.^29^ Many global public health gains made over the past two decades—mostly in relation to the MDGs and SDGs—were jeopardized due to the unprecedented situation presented by COVID-19, as reported by the United Nations.^30^ The COVID-19 pandemic worsened poverty, hunger, lack of clean water and sanitation, and unequal access to education. This threatens the fragile progress that has been made toward making societies more sustainable.^31^

**Figure 1:**
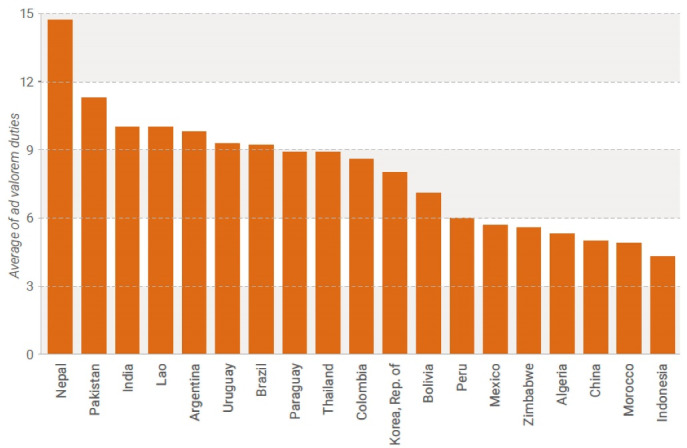
The world’s highest medicine tariffs. Source: WTO Tariff Database (http://tariffdata.wto.org)

 As many nations struggled to achieve UHC, the COVID-19 pandemic continued to exert enormous pressure on global health systems.^32^ Access to essential healthcare services is the lynchpin of health systems to fight against COVID-19.^33^ Maintaining the availability, provision, and use of basic healthcare services during the COVID-19 pandemic have been among the top concerns for many countries and international organizations in order to reduce morbidity and mortality.^34^ According to the WTO, the highest average tariff rates on imported medicines are found in South Asia and Latin America^35^ (Figure 1).

 Additionally, everyone needs to have access to affordable, safe, and effective COVID-19 vaccines, especially for the population that needs them the most, like frontline healthcare workers, as well as the disease’s diagnostic and treatment options. Against this background, GHD has been put into action. Several international organizations such as United Nations, WTO, World Bank, WHO, GAVI, COVAX, and UNICEF have played their constructive role through the GHD to make Access to Medicine for all. Moreover, realizing the gravity of the situation, TRIPS temporarily waived off the proposal on the part of India and South Africa submitted to the WTO Council. Now, the question is: Whether GHD left any positive impacts on SDG3, particularly in improving the ATM? The answer to this question is despondent, substantiated by the following facts. The COVID-19 vaccine, however, has not been distributed equitably. Worldwide, around 5.44 billion doses of COVID-19 vaccine have been administered as of September 1, 2021.

 Additionally, 3.15 billion individuals- 63.5% of whom resided in high-income nations and 1.8% in low-income nations—had at least one dose of the COVID-19 vaccine. In addition, 2.15 billion people worldwide received the full COVID-19 vaccine, with 53.3% of those individuals living in high-income countries and only 0.6% in low-income ones. Notwithstanding the GHD in place by several national and international players, the COVID-19 crisis has revealed significant ATM disparities and thereby exacerbating the existing inequalities both within and between developed and developing countries. It will jeopardize achieving SDG-3 in many contexts over time. Given this scenario, it is urgent to implement the recommendations (Table 3) of the Geneva network on the ATM if the governments are serious about achieving the SDGs for which GHD plays a pivotal role.

**Table 3:** Suggested low-cost steps governments could initiate to improve access to care

	**Reduce unnecessary medicine costs**	**Accelerate access to medicines**
Activities	1. Reducing taxes 2. Abolishing tariffs 3. Eradicating other trade barriers	1. Speeding up patent examination 2. Simplifying the drug approval process 3. Modernising government medicine reimbursement decision-making 4. Promoting open trade in medicines

Source:Geneva Network Report.^35^

## Conclusion

 Health as a human right obligates the states to ensure access to timely, acceptable, and affordable health care, in which access to essential medicines is essential. A joint statement by 15 think tanks from 14 countries recommended the states/governments might ensure ATM through two pathways: (1) reducing unnecessary medicines by reducing taxes, abolishing taxes, and eradicating other trade barriers and (2) accelerating ATM by speeding up patent examination, simplifying the drug approval process, modernizing government reimbursement decision making and promoting open trade in medicines. However, these pathways need global and regional collaboration, often negotiated at multilateral forums and organizations such as the WTO. Multilateral collaboration and partnerships could be built through health diplomacy at global and regional levels. In the recent past, African Medical Agency has emerged out of health diplomacy and continued and reinvigorated states’ engagement to contribute to the global public good, in this case, ATM.

## Acknowledgements

 The researchers thank Global Health Research and Innovations Canada team for providing coordination and administrative support for “ATM through GHD” literature review project.

## Competing Interests

 Vijay Kumar Chattu is one of the editorial board members of *Health Promotion Perspectives*. He is also the President and CEO of Global Health Research and Innovations Canada Inc. Other authors declare no competing interests.

## Ethical Approval

 Not applicable.

## Funding

 This research received no external funding.
